# Centrosome-associated regulators of the G_2_/M checkpoint as targets for cancer therapy

**DOI:** 10.1186/1476-4598-8-8

**Published:** 2009-02-13

**Authors:** Yingmei Wang, Ping Ji, Jinsong Liu, Russell R Broaddus, Fengxia Xue, Wei Zhang

**Affiliations:** 1 Tianjin General Hospital, Tianjin Medical University, Tianjin 300052, PR China; 2 Department of Pathology, The University of Texas MD Anderson Cancer Center, Houston, Texas 77030, USA

## Abstract

In eukaryotic cells, control mechanisms have developed that restrain cell-cycle transitions in response to stress. These regulatory pathways are termed cell-cycle checkpoints. The G_2_/M checkpoint prevents cells from entering mitosis when DNA is damaged in order to afford these cells an opportunity to repair the damaged DNA before propagating genetic defects to the daughter cells. If the damage is irreparable, checkpoint signaling might activate pathways that lead to apoptosis. Since alteration of cell-cycle control is a hallmark of tumorigenesis, cell-cycle regulators represent potential targets for therapy. The centrosome has recently come into focus as a critical cellular organelle that integrates G_2_/M checkpoint control and repairs signals in response to DNA damage. A growing number of G_2_/M checkpoint regulators have been found in the centrosome, suggesting that centrosome has an important role in G_2_/M checkpoint function. In this review, we discuss centrosome-associated regulators of the G_2_/M checkpoint, the dysregulation of this checkpoint in cancer, and potential candidate targets for cancer therapy.

## Introduction

With the aging of the world's population, the westernization of diet, and the increasing environmental pollution associated with the global economy, cancer has emerged as the top threat to human life worldwide [1,2]. To advance our progress against this disease, the two most important goals for cancer researchers are to fully understand the molecular basis of cancer and to develop effective therapies for it. One of the hallmarks of carcinogenesis is dysregulation of the cell cycle [3]. Cell cycle is controlled at a number of checkpoints. When cells suffer extracellular or intracellular stress or both, the cell-cycle checkpoints, especially G_1_/S and G_2_/M checkpoints which are controlled by a number of complexes that are composed of cyclin-dependent kinases (Cdks), cyclins, and their negative regulators including the Cip/Kip family members and the INK4a/ARF family members [4-6], are activated. The G_1_/S checkpoint is the first surveillance system to stop DNA synthesis when cells suffer from extracellular stresses and it is an effective step to control cell proliferation and apoptosis. The mechanism of G_1_/S checkpoint is extensively studied [5-8]. The G_2_/M checkpoint prevents DNA-damaged cells from entering mitosis and allows for the repair of DNA that was damaged in late S or G_2 _phases prior to mitosis. The G_2_/M checkpoint is controlled by Cdc2/cyclinB, and their negative regulators including p21^Cip1 ^and p27 [9]. Weakened G_2_/M checkpoint under therapeutic setting may trigger cell death via mitotic catastrophe for cells with unrepairable DNA lesions and mitosis machinery. This may represent a novel strategy to kill cancer cells, especially those with the p53 mutant phenotype which could result in inactivation or lost of the G_1_/S checkpoint in cancer [10,11]. Thus, the G_2_/M checkpoint is a potential target for cancer therapy.

As the primary microtubule-organizing center (MTOC), the centrosome plays an important role in maintaining chromosome stability by establishing bipolar mitotic spindles. Accumulating evidence suggests that centrosome integrates cell-cycle arrest and repair signals in response to genotoxic stress [12]. A growing number of important cell cycle regulators such as Cdks, checkpoint kinases (Chks), polo-like kinases (Plks), Aurora kinases, NIMA-related kinases (Neks), p53, BRCA1, and cyclin B1 have been shown to localize to the centrosome (Table 1). All of those proteins have been implicated in participating in G_2_/M checkpoint control and in the regulation of centrosome separation [13-20]. Abnormal expression (either under or over) of these proteins has been observed in most cancers [21] and they have been found to directly influence the efficacy of antitumor agents [22]. Thus, manipulating these G_2_/M checkpoint proteins could enhance cancer's sensitivity to radiotherapy and chemotherapy. In this review we focus on centrosome-associated regulators of G_2_/M checkpoint and potential targets for cancer chemotherapeutic therapy.

**Table 1:** Centrosome-associated G2/M checkpoint proteins

Centrosome proteins	Substrates	Functions	Effects of expression manipulation
cyclin B/Cdk1 [33]	Drp1/Dnml1, HuR, hnRNP-k, TPX2	mitosis entry, bipolar spindle assembly	inhibition: induce cell cycle arrest and apoptosis
Aurora A[34,35,76]	centrosomin, γ-TuRC, Eg5, Ran-TPX2, CENP-A, PP1, p53, Cdh1, NM23-H1, CPEB, Cdc25B, TPX2	mitotic entry and exit, centrosome mutation and separation, spindle formation	inhibition: monopolar spindle overexpression: centrosome amplification and loss of mitotic checkpoint
Aurora B[34,35]	INCEP, Survivin, BubR1, Mad2	chromatid separation, spindle assembly checkpoint	inhibition: multinucleate cells
Plk1[21,34,36]	Cdc25, cyclinB/Cdk1, p53, Nlp1, ATM/ATR, BRCA1, Chk1, Emi1, Wee1	mitotic entry and exit, APC/C regulation, bipolar spindle formation, centrosome maturation,	inhibition: smaller centrosomes
Nek2A[18,34,101]	PP1, C-Nap1	centrosome separation and maturation, mitotic entry	overexpression: split centrosomes
Survivin[90,91,102]	Caspases 3, 7, 9, Aurora B, INCENP	anti-apoptosis	inhibition: loss of mitotic kinases and checkpoint, supernumerary centrosome
p53[47,48]	p21, 14-3-3, GADD45	centrosome duplication	inhibition: centrosome amplication
BRCA1[51,52]	γ-Tubulin, Chk1/2, p53, Cdc25, Wee1, Aurora A	centrosome duplication	inhibition: centrosome re-duplication and hyperactive MT nucleation
APC/C[99]	Cyclin B/Cdk1, securin, Aurora A, Plk1, Cdk2	sister chromatid separation, mitotic exit, proteasomal degradation	NA
ATM/ATR[55]	p53, Chk1/2, BRCA1, Mdm2	initiation of genotoxic stress response	NA
Chk1/2 [56-59]	Cdc25, BRCA1, E2F, p73α	centrosome separation, mitotic entry	inhibition: centrosome amplification and mitotic arrest

### Cell cycle and centrosomal cycle

The cell cycle entails a recurring sequence of events that include the duplication of cellular contents and subsequent cell division. Traditionally, the cell cycle in the eukaryotic cell is divided into four phases: Gap phase 1 (G_1_); DNA synthesis phase (S); Gap phase 2 (G_2_), during which the cell prepares itself for division; and mitosis phase (M), during which the chromosomes separate and the cell divides. The M phase includes prophase, metaphase, anaphase, and telophase [23].

Centrosome, the nonmembranous organelles that occupy a tiny volume near the center of the cell, are usually proximal to the nucleus. In most vertebrate cells, the centrosome is classically depicted as having two orthogonally positioned cylindrical centrioles surrounded by a matrix of fibrous and globular proteins that constitute the pericentriolar material (PCM)[24]. The cell cycle involves an intricate process of DNA replication and cell division that concludes with the formation of two genetically equivalent daughter cells. In this progression, the centrosome is duplicated only once to produce the bipolar spindle and ensure proper chromosome segregation. Centrosome maturation and separation are tightly regulated during the cell cycle. Centrosome duplication consists of the five morphological steps during cell cycle progression [25]. 1) In early G_1_/S phase, the mother and daughter centrioles separate slightly and lose their orthogonal orientation; 2) in S phase, synthesis of a daughter centriole occurs in the vicinity of each preexisting centriole; 3) in G_2 _phase, the procentrioles elongate to complete the duplication process. The duplicated centrosome disjoins into two functionally separate centrosome, each containing a mother-daughter pair of centrioles; 4) in late G_2 _phase, the centrosome increases in size and separate to allow the formation of a bipolar spindle; 5) in M phase, the original mother and daughter centrioles detach from each other in an event termed centrosome disjunction. Since centrosome duplicates only once during the normal cell cycle, duplication of centrosome must proceed in coordination with DNA synthesis to synchronize with cell division [26]. Centrosome appears to be a critical organelle for G_2_/M checkpoint. Centrosome separation is initiated at the G_2 _phase and completed in the M phase. Several key proteins involved in controlling the G_2_/M checkpoint have been shown to physically associate with centrosome.

### Centrosome-associated regulators of G_2_/M checkpoint

**Figure 1:**
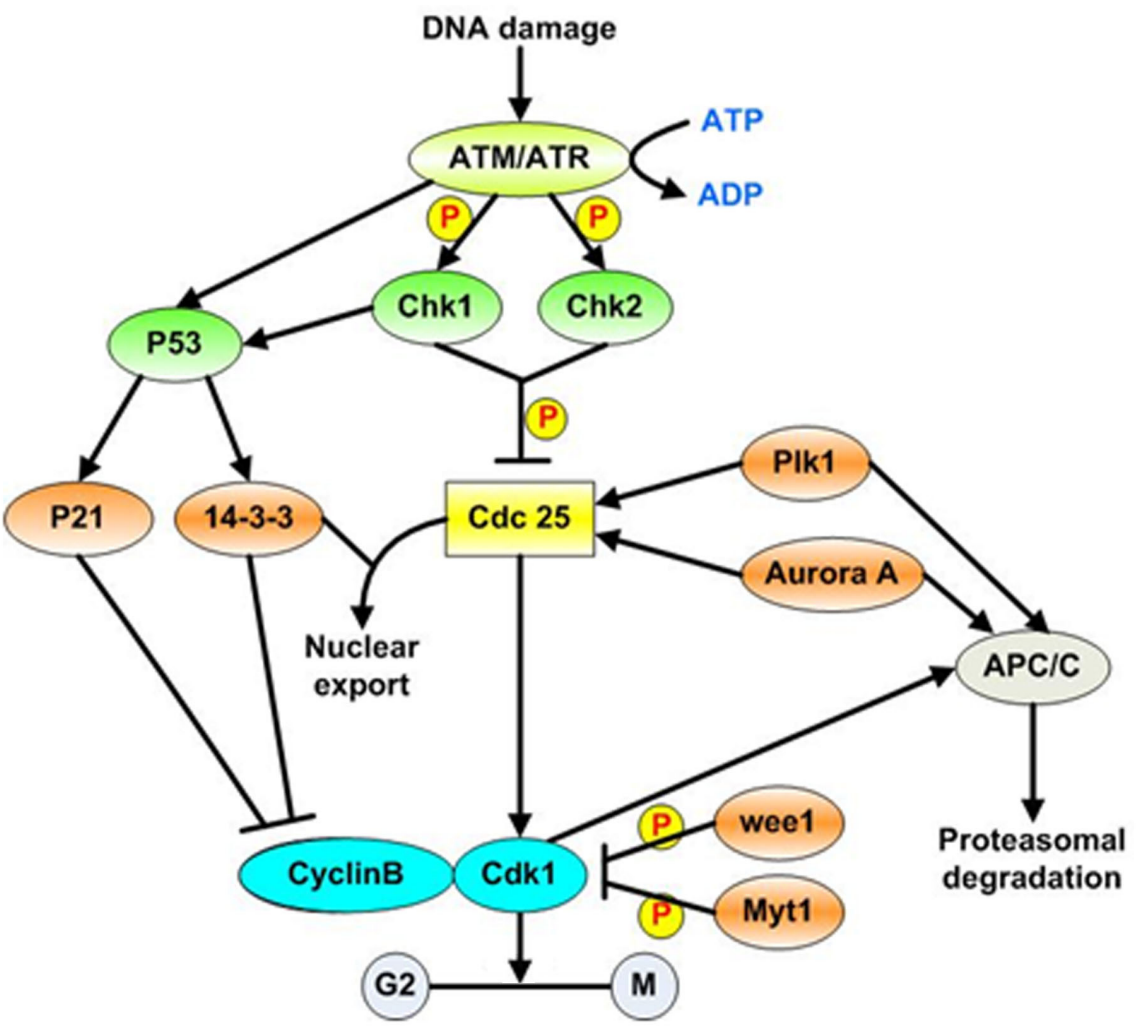
**Activation of the G2/M checkpoint after DNA damage**. In response to DNA damage, the ATM, ATR signaling pathway is activated, which leads to the phosphorylation and activation of Chk1 and Chk2 and to the subsequent phosphorylation of Cdc25. Phosphorylated Cdc25 is sequestered in the cytoplasm by 14-3-3 proteins, which prevents activation of cyclinB/Cdk1 by Cdc25 and results in G2 arrest. Activated ATM/ATR also activates p53-dependent signaling. This contributes to the maintenance of G2 arrest by upregulating 14-3-3, which sequesters Cdk1 in the cytoplasm. In addition, p53 induces the transactivation of p21, a Cdk inhibitor that binds to and inhibits cyclinB/Cdk1 complexes. P: phosphorylation.

An increasingly number of cancer related proteins have been shown to reside in or traffic in and out of centrosomes. These regulators include: 1) A number of cell cycle-regulated proteins, including cyclin B1, Cdks, Chks, Plks, aurora kinases, and Neks [27-36]; 2) Oncogenes, such as Survivin, Ras, Rad6, and HER2/neu [37-40]; 3) Tumor suppressors including p53, Rb, p21, XRCC2/3, APC, NM23-R1/H1, Gadd45 and BRCA l/2 [19,20,41-48]; and 4) Ubiquitination and degradation related proteins, including anaphase-promoting complex/cyclosome (APC/C), BRCA1, Cdc20, and Cdh1 [49-52]; 5) DNA damage checkpoint proteins including ATM, ATR, p53, BRCA1, Chk1, and Chk2 [29,53-59]. More detailed information about these regulators is listed in Table 1. The roles of these centrosome-associated regulators have been extensively investigated and some of the current understanding of their roles in G_2_/M checkpoint and in response to DNA damage is summarized in Fig 1. In this section, we will review the regulatory roles of the key centrosome-related kinases and some cancer related genes involved in G_2_/M transition.

Cdc2 (also termed Cdk1) and its regulator cyclin B drive cells into mitosis from G_2 _phase. In early G2 phase, Cdk1 is inactivated by phosphorylation of T14 and Y15 residues by Wee1 and Myt1 kinases [60]. The initial activation of cyclin B/Cdk1 occurs at the centrosome in prophase. This involves Cdk1 dephosphorylation at T14 and Y15 by Cdc25 phosphatase family (Cdc25A, B, and C) and cyclin B phosphorylation at Ser126/128 by MPF and Ser133 by Plk1 [61-65].

Chk1 and Chk2 are transducers of ATR- and ATM-dependent signaling in response to DNA damage. Chk1 has been detected at the interphase centrosome, and inhibition of Chk1 resulted in premature centrosome separation [29]. Chk2 was also reported to localize to the centrosome and could be phosphorylated at Thr 68/26 and Ser 28 by Plk1, which co-localized with Chk2 at the centrosome in early mitosis [66]. Chk1 is activated by ATR in cells treated with ultraviolet radiation [67], whereas Chk2 is activated by ATM in cells exposed to ionizing radiation [68]. Activation of ATM/ATR initiates the subsequent protein kinase cascade through both p53 dependent and independent pathways. In p53 dependent pathways, p53 is phosphorylated on Ser 15 and Ser 20 [69-71] and then activates downstream targets genes, such as p21 and 14-3-3, which play an important role in G_2_/M checkpoint through inhibition of Cdk1/cyclin B [72,73]. In the p53 independent pathway, Chk1 and Chk2 phosphorylate Cdc25 at Ser 216, which down-regulate Cdc25 activity by promoting 14-3-3 protein and nuclear export [53,61]. Chk1/2 also phosphorylates Wee 1 and increases Wee 1 activity. It is known that both Cdc25C and Wee 1 phosphorylation cooperatively reduce Cdk1/cyclin B1 activity leading to G_2_/M arrest [61,74,75].

In mammalian cells, three members of the Aurora family have been identified: Aurora A, B, and C. Among them, Aurora A is associated with the centrosome and microtubules. Aurora A is essential for controlling multiple steps in the cell cycle from late S phase through M phase, including centrosome maturation and separation, mitotic spindle formation, and mitotic entry and exit. Aurora A mediates its multiple functions by interacting with other centrosome proteins including p53, centrosomin, centromere protein A, Eg5, and BRCA1[76].

Plk1, which is the best studied member of the Plk family in mammalian cells, is involved in various events in mitotic progression [77]. Plk1 increases during S and G_2_/M [78]. Plk1 phosphorylates and activates Cdc25, which leads to activation of Cdk1/cyclin B1 and G_2_/M checkpoint [79,80]. Plk1 also plays a role in mitosis exit by regulating the anaphase-promoting complex [81,82]. In response to DNA damage, Plk1 activity is inhibited in an ATM/ATR dependent manner [83], preventing mitosis entry.

Nek2, which is a member of the Nek kinase family, has a role in regulation of the G_2_/M checkpoint and is localized to the centrosome. Nek2 has two splice variants: Nek2A and Nek2B. Nek2A is required for centrosome separation at the G_2_/M transition and forms a complex with the catalytic subunit of protein phosphatase 1 (PP1) and a large coiled-coil protein called C-Nap1 [84,85]. Nek2 can phosphorylate its substrates, C-Nap1 and Nlp, contributing to their displacement from the centrosome, which is an essential step for subsequent splitting of the centrosome [85-87].

Survivin is a member of the inhibitor of apoptosis protein (IAP) family that plays an essential role in the control of cell division and the inhibition of apoptosis [88]. Survivin is expressed in a cell cycle-dependent manner and regulates G_2_/M phase by localizing to multiple sites on the mitotic apparatus including the centrosome, microtubules, and the mitotic spindle [37]. Also, Survivin performs its mitotic roles by cooperating with inner centromere protein (INCENP) and Aurora B [89]. A basic event for Survivin regulation is phosphorylation of the Thr34 by the p34(Cdc2) kinase [90,91]. Survivin induces apoptosis by inhibiting, directly or indirectly, the activity of Caspases 3, 7, and 9.

Accumulating evidence indicates that BRCA1 is located in the centrosome and binds to γ-tubulin [92]. BRCA1 has an important role in regulating centrosome duplication. This tumor suppressor is involved in all phases of the cell cycle and regulates orderly events during cell-cycle progression through its transcriptional activity and ubiquitination ligase E3 function [92-95]. BRCA1 interacts with many proteins that play important roles in multiple biological pathways. These proteins include ATM, ATR, Chk1/2, Wee1, p53, Aurora A, and Cdc25C [93,94,96,97], all of which have important roles in G_2_/M cell cycle regulation.

The ubiquitin-proteasome pathway (UPP) is essential for degrading intracellular proteins, which plays a key role in maintaining cellular homeostasis. Polymers of ubiquitin are covalently attached to protein targets by three key enzymes: ubiquitin-activating enzyme E1, ubiquitin-conjugating enzymes E2, and ubiquitin ligases E3. The resulting ubiquitinated proteins are then recognized and degraded by the 26S proteasome [98]. Cyclin B/Cdk1 is a master regulator during G_2_/M transition, and cyclin B/Cdk1 activity is strictly governed by the anaphase-promoting complex/cyclosome (APC/C), a ring-finger-type E3 that plays an important role in sister chromatid separation and exit from mitosis by degrading mitotic substrates. The APC/C is activated by its adaptor and regulators, such as Cdc20 and Cdh1, to target Securin and mitotic cyclins. Activation of APC/C is required for anaphase onset and mitotic exit [99].

### Dysregulation of the centrosome-associated regulators of G_2_/M checkpoint in cancer

Mounting evidence indicates that cell-cycle dysregulation is a common feature of cancer. The G_2_/M checkpoint in particular is an area of focus for cancer research. Abnormalities of several of above mentioned centrosome-associated regulators of the G_2_/M checkpoint have been detected in human tumors, as detailed below [21,100-102]:

The Aurora A gene is located on chromosome 20q13.2, a region that is commonly amplified in many epithelial cancers. Both mRNA and protein levels of Aurora A are overexpressed in a variety of tumor tissues and tumor cell lines, suggesting its potential role in tumorigenesis. Aurora A mRNA upregulation has been significantly associated with advanced tumor stage, the presence of positive regional lymph nodes, as well as distant metastasis in head and neck squamous cell carcinoma [100]. Aurora A also promotes cell migration and reduces the radiosensitivity of laryngeal squamous cell carcinoma [103]. In ovarian cancer, overexpression of Aurora A is associated with centrosome amplification and poor survival [104]. Overexpression of Aurora A was significantly associated with aggressive clinical behavior including high histologic grade, invasion, metastasis and overall survival of patients with bladder cancer. Aurora A gene copy number has been reported to be a promising biomarker for detection of bladder cancer [105,106].

Plk1 expression has been showed to be elevated in non-small-cell lung, head and neck, esophageal, gastric, breast, ovarian, endometrial, colorectal, and thyroid carcinomas; melanomas, and gliomas [21]. Overexpression of Plk1 correlates positively with tumor stage, nodal status, and diffuse growth pattern in human gastric cancer [107]. In a study of 158 colon cancer patients, Weichert et al. found that overexpression of Plk1 correlated positively with Dukes stage and nodal status [108].

Overexpression of active Nek2A kinase leads to premature splitting of the mother and daughter centrioles [18,109], whereas expression of inactive Nek2A kinase causes the formation of centrosomal abnormalities, monopolar spindles, and aneuploidy [110], all of which are involved in regulating genetic stability and tumorigenesis. Elevated protein expression of Nek2 leads to centrosome abnormality and, consequently, tumorigenesis. Nek2 expression is elevated in breast, ovary, cervical, prostate cancers, and leukemia [101].

Abnormal expression of Survivin in mammalian cells could result in aberrant mitotic progression characterized by cell division defects that include supernumerary centrosomes, mislocalization of mitotic kinases, and loss of mitotic checkpoint. Survivin is overexpressed in a wide spectrum of human cancer, including lung, breast, colon, gastric, liver, bladder, uterine, and ovary cancer [102].

Heat-shock protein 90 (hsp90), a molecular chaperone, plays a role in G_2_/M checkpoint regulation by associating with its client proteins including Chk1, Cdk1, Wee1, Myt1, Plk1, and cyclinB through regulation of their stability. Hsp90 inhibitors could result in targeting of these client proteins to the proteasome to be degraded which may explain the substantial G_2_/M peak in cell cycle [111-114].

The APC/C, a multisubunit ubiquitin ligase E3, is a gatekeeper for mitosis by balancing the amount of checkpoint regulators. Two key activators for APC/C function are Cdh1 and Cdc20. Dysfunction of APC/C^Cdh1 ^might result in abnormal accumulation of both mitotic Cdk activity and non-Cdk kinases activity(such as Aurora A, Plk, and Nek2), leading to the development of cancer [115]. APC/C^Cdc20 ^recognizes and marks the key substrate securin and cyclin B1 for degradation and promotes chromosome separation and anaphase onset in a time- and spatial-dependent manner. Deregulation of Cdc20-dependent proteolysis can result in aneuploidy, ultimately resulting in cancer. Securin has been reported to be overexpressed in human breast and colorectal cancers [116,117]. In addition, Hagting et al. found that blocked proteolysis of securin by APC/C^Cdc20 ^led to genomic instability in cultured cells [50]. Thus, dysfunction of the APC/C might lead to uncontrolled proliferation, genomic instability, and cancer.

### Modulation of G_2_/M checkpoint proteins and cancer therapy

Although there are defects in G_2_/M checkpoint proteins in cancer, the nature of these alterations is quite different from that of alterations of the G_1_/S checkpoint. The presence of p53 mutation in 50% of all cancers renders the G_1_/S checkpoint less efficient, allowing synthesis of unrepaired DNA [11,118]. For G_2_/M checkpoint proteins, mutations of key players are not common. Even for BRCA1, mutation is infrequent in sporadic cancers and more concentrated in the familial breast cancers [94]. The impact of p53 as a checkpoint protein is complex because p53 is also a major regulator of apoptosis [119]. Because cell cycle checkpoints also repair DNA damages caused by therapeutics, the role of cell cycle checkpoints are often the cause for resistance. On one hand, increased proliferation is a common feature for aggressive cancers, thus inhibition of cell proliferation is a logical approach. On the other hand, most cancer drugs target cycling cells, so the fast growing tumor cells are more sensitive to these treatments. It is well-known that slow-growing and more differentiated cancers are generally resistant to chemotherapy. As a matter of fact, the G_2_/M checkpoint is invariably activated in cancer cells in response to DNA damage partially causing resistance to therapy [120,121]. Specifically, the G_2_/M checkpoint based anti-cancer strategies have been focused on targeting and inactivating the G_2_/M checkpoint, thus forcing the cancer cells into mitosis with increased DNA damage and finally into mitotic catastrophe and cell death. Following is a brief review on some of the checkpoint related cancer therapies under development [122-127] (Table 2).

**Table 2:** Small molecule inhibitors targeting centrosome associated G_2_/M checkpoint regulators

	Specific targets	Functions	Clinical development	Combination with DNA-damaging agents
Flavopiridol[128]	Cdk1, p21, Survivin	Bind to Cdc2, inhibit apoptosis	Phase I/II	paclitaxel, irinotecan, gemcitabine, IR
UCN-01[153-155]	Cdk1, Chk1/2	Inhibit Chk1 and PKC activity, promote apoptosis	Phase I/II	fluorouracil, topotecan, cisplatin, IR, temozolomide
Daidzein[139]	Cdk1, p21^Cip1^, p57^Kip2^	Inhibit Cdk1 and cell proliferation	Preclinical	NA
Caffeine [123-125]	ATM/ATR, PI3K	Inhibit ATM/ATR, cause G2 checkpoint abrogation, induce apoptosis	Phase I	Taxol, cisplatin
KU-55933[122,126]	ATM	Inhibit ATM	Phase I	NA
Berberine[140,141]	Wee1, 14-3-3, Cdk2, cyclinB, Cdc25c	Induce G2/M phase arrest and apoptosis	Preclinical	NA
17AAG [145-147]	Chk1, Hsp90	Downregulate Chk1, inhibit colony formation, induce apoptosis, abrogate G2/M checkpoint	Phase I/II	gemcitabine, cisplatin, topoisomerase I poisons, taxol, IR
XL844[148]	Chk1/2	Enhance gemcitabine antitumor activity	Phase I	Gemcitabine
CHIR-124[149]	Chk1	abrogate G2/M checkpoint, induce apoptosis	Preclinical	Topoisomerase I poisons
PF-00477736[150]	Chk1, cyclin B, Securin, Aurora	Inhibit Chk1, abrogates cell cycle arrest	Preclinical	Gemcitabine, carboplatin
PD0166285[127]	Wee1, Aurora A	Inhibit Wee1, abrogate G2/M checkpoint	Preclinical	NA
CEP-3891[143,151]	Chk1	Abrogate G2/M checkpoint	Preclinical	IR
N-aryl-N'-pyrazinylurea [142,152]	Chk1	Inhibit Chk1, inhibit cell proliferation	Preclinical	Doxorubicin, camptothecin
VX-680[166,170]	Aurora A, B	Block cell proliferation, disrupt bipolar spindle formation, accumulate of cell with 4N or greater DNA	Phase II	Vorinostat, docetaxel
Hesperadin[171]	Aurora kinase	Inhibit Aurora kinase activity	Phase I/II	NA
ZM447439[173]	Aurora A, B	Inhibit Aurora A, induce apoptosis	Phase I	NA
PHA-739358[164]	Aurora A, B, C	Inhibit proliferation	Phase I	NA
PHA-680632 [167,163]	Aurora A	Inhibit Aurora A and proliferation	Phase I	IR
ON01910[179]	Plk1, Cdk1	Inhibit Plk1, induce mitosis arrest	Preclinical	NA

Abbreviation: NA, not available; IR, ionizing radiation

### Cdc2 inhibitors

To date, the majority of the published data suggests that inhibition of cyclin/Cdk complexes may prevent or delay tumor progression in cancer patients. Among a number of Cdk inhibitors under development, flavopiridol and UCN-01 are being tested in clinical trials [3,128]. We will review flavopiridol as an example.

Flavopiridol binds and directly inhibits Cdc2 as well as inhibiting antiapoptotic molecules including p21, Bcl2, and Survivin [129-131]. Flavopiridol has been tested as a novel chemotherapeutic agent for rhabdoid tumors, osteosarcoma, Ewing's family tumor cells, and leukemia [131-133]. The combinations of flavopiridol with paclitaxel, irinotecan, or gemcitabine have shown promising effects in cell line studies and in clinical trials. It was reported that paclitaxel or docetaxel followed by flavopiridol is associated with an increased induction of apoptosis through accelerating exit of cells from mitosis, but the reverse treatment schedule did not show added effect than paclitaxel or docetaxel alone [134-136]. Recently, it was reported that paclitaxel treatment followed by carboplatin for 1 hour and flavopiridol over 24 hours every 3 weeks for 3 cycles was effective and safe in NSCLC patients [137]. A greater antitumor effect was observed with the combination of gemcitabine or irinotecan followed by flavopiridol in several epithelial gastrointestinal cell lines [129,138]. Thus, flavopiridol in combination with chemotherapy may overcome cell cycle mediated drug resistance.

Other regulators of cyclin/Cdk complexes and Cdk inhibitors have been reported. Treatment with the isoflavone daidzein decreased the expression of Cdc2 and increased the expression of the Cdk inhibitors p21^Cip1 ^and p57^Kip2 ^in MCF-7 and MDA-MB-453 cells. Thus, daidzein exerts its anticancer effects in human breast cancer cells via cell-cycle arrest [139]. Berberine has been reported to induce G_2_/M arrest in leukemia and gastric cancer cells via the inhibition of cyclin B1 and the promotion of Wee1 [140,141].

### Chk1 inhibitors

There are a large reservoir of identified Chk1 inhibitors including UCN-01, 17AAG, XL844, CHIR-124, PF-00477736, CEP-3891, and N-aryl-N'-pyrazinylurea. UCN-01, 17AAG, and XL844 are being tested in clinical trials, while the others are still in preclinical studies [142-152]. UCN-01 has been reported to promote apoptosis through G_2_/M checkpoint abrogation in various human cell lines. Thus, UCN-01 exerts more marked antitumor effects through combination with radio- or chemotherapy [153-155]. Results of three Phase I studies of combination therapy with UCN-01 in patients with solid tumors have been published, in which UCN-01 was combined with fluorouracil [156], topotecan [157], and cisplatin [158], respectively. UCN-01 plus topotecan or carboplatin were found to be generally well tolerated; however, combination of UCN-01 and fluorouracil did not show significant antitumor activity against advanced ovarian cancer [159,160]. Further research to develop these combinations is warranted, especially focusing on reducing side effects.

### Aurora Kinase Inhibitors

The evidence linking Aurora kinase overexpression and malignancy has stimulated interest in identifying and developing Aurora kinase inhibitors for cancer therapy. RNA interference targeting Aurora A has been found to suppress tumor growth and enhance sensitivity to chemotherapy- and radiation-induced apoptosis in human cells [161,162]. Several Aurora kinase inhibitors, including VX-680, Hesperadin, ZM447439, AT-9283, MLN-8054, R-763, SU6668, and PHA-739358, have been identified and are undergoing phase I/II clinical trials [163-167].

One of these inhibitors, VX-680, the first Aurora kinase inhibitor to enter clinical trials, not only inhibits cell proliferation but also induces apoptosis in a wide spectrum of tumor types. VX-680 was shown to greatly inhibit tumor growth *in vivo *in three xenograft models of leukemia, colon, and pancreatic tumors. It was reported that VX-680 has no effect on non-cycling normal cells which makes it a promising anticancer agent [168]. VX-680 also was found to be effective in reducing cell growth in different anaplastic thyroid cancer-derived cell lines [169]. In ovarian cancer, combination of VX-680 (also known as MK-0457) with docetaxel could significantly reduce cell proliferation and increase tumor cell apoptosis than VX-680 or docetaxel alone *in vivo *[170]. Further investigation of this inhibitor is warranted to exploit its potential value in the treatment of cancer.

In tobacco BY-2 cells, another Aurora kinase inhibitor, Hesperadin, was found to induce delayed transition from metaphase to anaphase and early exit from mitosis after chromosome segregation [171]. It is not clear, however, whether Hesperadin causes tumor cell death. In a colony formation assay, ZM447439, another Aurora kinase inhibitor, was found to be more toxic to proliferating cells than to nondividing cells [172], indicating that it might also be used selectively to kill proliferating tumor cells. ZM447439 is an effective apoptosis-inducing and G_2_/M phase-arresting agent in acute myeloid leukemia and Hep2 carcinoma cells [173,174].

### Inhibitors of Plk1

The G_2_/M phase regulator Plk1 is frequently overexpressed in cancers and correlates with aggressiveness and poor prognosis. Cogswell *et al *observed that silencing of Plk1 functions induced apoptosis accompanied by mitotic catastrophe in SAOS-2 and U-2OS tumor cells but not in normal human mammary epithelial cells [175]. Findings from another study suggested that reduction of Plk1 expression via small interfering RNAs (RNAi) could prevent the growth of bladder cancer *in vivo *[176]. Downregulation of Plk-1 expression by RNAi has been found to cause cell-cycle arrest at the G_2_/M phase, reduce cellular proliferation, and increase gemcitabine cytotoxicity in pancreatic tumor cells *in vitro *[177].

Small molecule inhibitors of Plk1 include ATP-competitive and non-ATP-competitive categories. Identifying specific ATP-competitive inhibitors is challenging because of the high degree of structural conservation among ATP-binding domains in various kinases [178]. ON01910, a non-ATP-competitive Plk1 inhibitor, was reported to inhibit cancer cells growth by inducing mitosis arrest and apoptosis in many tumor cell lines. Importantly, ON01910 did not show hematotoxicity, liver damage, or neurotoxicity *in vivo *[179]. Thus, ON01910 is a promising Plk1 inhibitor that may exhibit beneficial effect in patients.

### Summary and future directions

Cell cycle checkpoints provide mechanisms for cells to repair DNA damage. Activated checkpoints slow down cell cycle progression and thus allow normal cells to repair damage to prevent propagation of damaged DNA. The development of anti-cancer therapeutics has capitalized on the fact that activation of checkpoint proteins results in attenuated cell proliferation lead to anti-growth cancer therapeutics. Drugs have been developed to arrest cancer cells and stop cancer cell proliferation. On the other hand, the same mechanism that normally protects cells from DNA damage also repairs DNA following chemotherapy and radiotherapy. Therefore, strategies have been developed to abrogate the checkpoint activation, and drugs that exert this effect are combined with chemo- or radiotherapy to enhance cell kill.

In addition to small molecule inhibitors, gene based therapeutics such as antisense oligonucleotides also show promise. Recently, there is growing interest in a class of small RNA termed microRNAs (miRNAs) [180]. The miRNAs are a class of small (~22 nucleotides) noncoding RNAs that functions as post-transcriptional gene regulators [181]. miRNAs may regulate the expression of many genes, such as tumor-suppressor genes and oncogenes as well as their molecular networks [182-186], which in turn impact cell cycle progression [187,188]. miRNAs regulate a wide range of biological processes, including cell differentiation, proliferation, and apoptosis [189]. Aberrant miRNAs expression is involved in human tumorigenesis [190,191]. Mertens-Talcott *et al *[184] demonstrated that miR-27a increased the percentage of MDA-MB-231 cells in G_2_/M by inducing its target gene Myt-1, which inhibits G_2_/M through enhanced phosphorylation and inactivation of Cdk1. Yang et al [185] showed miR-214 induces cell survival and cisplatin resistance primarily by down-regulation of PTEN protein and activation of the Akt pathway through 3'-untranslated region (UTR) of the PTEN in human ovarian cancer. According to Yang et al [192], let-7i expression was significantly reduced in chemotherapy-resistant epithelial ovarian cancer patients. The *in vitro *study showed that reduced let-7i expression significantly increased the resistance of ovarian and breast cancer cells to cis-platinum. Thus, it was proposed that let-7i could be targeted in platinum-resistance patients. Taken together, miRNAs emerge as new therapeutic targets as well as tools in cancer treatment.

Cancer stem cells (CSCs) have become a new focus in cancer research since they may play a role in cancer initiation, metastasis, treatment resistance, and recurrence [193]. CSCs have been found in hematopoietic cancers as well as solid tumors included brain, neck, lung, breast, liver, colon, pancreas, prostate, bone, and melanoma [193-199]. Investigations into characteristics of CSCs improved our understanding of tumor treatment resistance. Conventional chemo- or radiotherapies preferentially kill dividing cells, but CSCs are low-growing, which make them resistant to conventional therapy. It is also likely that conventional therapies actually enrich CSCs and these cells have to potential to repopulate. Therefore, failure to target CSCs predicts for cancer recurrence. Current studies on CSCs zero in on the limitless proliferative capacity, self-renewal pathways, drug efflux pumps, and their niche [200]. Whether and how these features are linked to cell cycle checkpoints are not clear although they will likely be linked. The development of strategies that target CSCs as well as checkpoint will likely crosses paths and has potential in emergence in a new class of highly effective cancer therapeutics.

## Competing interests

The authors declare that they have no competing interests.

## Authors' contributions

All authors have read and approved the final version of the manuscript. YMW drafted the manuscript and generated tables and figure. PJ, JL, RB and FXX contributed to the writing and editing of the manuscript. WZ contributed to the writing of the manuscript and supervised the project.
